# 2218: Investigating the correlation between rheumatoid arthritis and *Prevotella copri*

**DOI:** 10.1017/cts.2017.211

**Published:** 2018-05-10

**Authors:** Hannah Fehlner-Peach

## Abstract

OBJECTIVES/SPECIFIC AIMS: Rheumatoid arthritis (RA) is one of the most prevalent systemic autoimmune diseases. It is caused by a combination of genetic and environmental factors. In humans, the intestinal microbe *Prevotella copri* strongly correlates with RA in previously untreated new-onset rheumatoid arthritis (NORA) patients. Metagenomic assembly of *P. copri* from NORA patients and healthy controls suggests genetic differences between *P. copri* from each group. In order to test the hypothesis that genetic differences in *P. copri* from arthritis patients promote arthritis, I am performing genomic comparison of primary *P. copri* isolates from NORA patients and healthy controls, and analysis of the immune response to *P. copri* in mice. Mice colonized with *P. copri* have increased susceptibility to DSS-induced weight loss and death compared with uncolonized controls. Future experiments will assess the local and systemic immune response in *P. copri*-colonized, DSS-treated mice. If this work is successful, then it may be possible to exploit genetic variation in *P. copri*. This could lead to new biomarkers for human disease or even insight into drug metabolism. METHODS/STUDY POPULATION: To validate a strategy to screen for the presence of *P. copri* in feces, qPCR primers were designed to amplify 8 regions across the 3.5 Mb *P. copri* reference genome using NCBI PrimerBlast. Primers were validated with DNA from feces for which *P. copri* abundance was previously determined by 16S rDNA sequencing. *P. copri* genome-specific primers were used to screen bacterial isolates from NORA patients and healthy controls. The 16S V3-V4 region was sequenced and compared with the *P. copri* reference 16S sequence to confirm >97% similarity. Genomes of 2 NORA patient isolates were sequenced on Illumina MiSeq, and sequences were compared with the reference genome. A strategy was developed to colonize mice with *P. copri*: 3-week-old C57BL/6 mice were treated with antibiotics in drinking water for 2 weeks, then switched to water for 2 days before oral gavage with *P. copri*; 6–7 days after inoculation, *P. copri* colonization was assessed by plating feces from inoculated mice, and by qPCR of fecal DNA with *P. copri*-specific primers. A systemic immune response to *P. copri* was assessed by microbe-specific ELISA for IgG and IgA in the sera of colonized mice. RESULTS/ANTICIPATED RESULTS: *P. copri* was detected in the stool of 20% of healthy individuals and 50% of NORA patients. *P. copri* was isolated form 4 healthy individuals and 6 NORA patients. Whole genomes of 96 primary isolates from NORA patients and healthy controls will be sequenced on the Illumina HiSeq platform, and their genomes will be assembled and compared using Spades software. For 2 *P. copri* isolates for a NORA patient, 89% of 250 bp reads aligned >95% to the *P. copri* reference genome. Mice can be colonized with *P. copri* gavaged at >106 CFU. *P. copri*-specific IgG and IgA were detected in the sera of colonized mice. DISCUSSION/SIGNIFICANCE OF IMPACT: Several primary isolates of *P. copri* have been collected from healthy controls and NORA patients, which will enable whole genome comparison of these isolates. For the 2 *P. copri* isolates sequenced, 89% of 250 bp reads aligned >95% to the *P. copri* reference genome, indicating variability between NORA patient *P. copri* strains and the *P. copri* reference genome. The establishment of colonization of mice with *P. copri* will allow further characterization of the immune response to *P. copri* at steady state and under pro-inflammatory conditions. Further, the systemic immune response to *P. copri* indicates that this microbe may have potential to play a role in systemic disease.

